# Lifestyle Factors in the Clinical Manifestation and Management of Atopic Dermatitis

**DOI:** 10.26502/aimr.0193

**Published:** 2025-01-30

**Authors:** Kelly Lam, Devendra K. Agrawal

**Affiliations:** 1 Department of Translational Research, College of Osteopathic Medicine of the Pacific, Western University of Health Sciences, Pomona, California 91766 USA.

**Keywords:** Atopic dermatitis, Eczema, Inflammation, Lifestyle factors, Proinflammatory cytokines, Pruritus, Skin barrier

## Abstract

Atopic dermatitis (AD), also known as eczema, is an inflammatory dermatologic condition that results in inflamed, itchy skin lesions. The development of this condition is governed by a variety of genetic and environmental factors including lifestyle habits. The severity of atopic dermatitis has been attributed to be affected by various lifestyle factors, prompting the interest in utilizing lifestyle modifications as a form of treatment for atopic dermatitis symptoms. Many research studies have been conducted to investigate the effects of different factors such as sleep, stress, diet, smoking and tobacco use, exposure to various temperatures and humidity levels, and skincare and cosmetic products on atopic dermatitis symptoms, and how certain habits can be modified to manage AD conditions. Current studies have demonstrated the significant impact some lifestyle modifications can elicit with improving atopic dermatitis, while also discussing other lifestyle factors that require further research to determine their effects on AD. This review article summarizes the findings in the current literature that investigates the role of different lifestyle habits on the severity and exacerbation of atopic dermatitis, and explores the mechanisms in which these behaviors can trigger AD.

## Introduction

Atopic dermatitis (AD) is a chronic inflammatory dermatologic disorder negatively impacting up to 2.6% of the global population [1]. Also known as ‘eczema,’ atopic dermatitis is generally characterized by recurring erythematous, pruritus, lichenified lesions [2]. The clinical presentation of atopic dermatitis varies depending on the severity of the condition. In more mild cases, AD presents with indistinct erythema, slightly raised epidermis, superficial excoriations, and faint lichenification [3]. Moderate cases of AD present with more visible erythema, noticeable edema, deeper excoriations, and obvious lichenification with markings [3]. In severe AD, erythema is deep and dark, visible plaques and raised papules are present and are accompanied with deep and dense excoriations [3]. Lichenification in severe AD is thick with deep markings [3]. When assessing the clinical manifestations of AD, it is important to consider the presentation variations found in lighter and darker pigmented skin.

The development of atopic dermatitis is governed by a variety of factors including associations between genetic and environmental components [4]. Additionally, there are ethnic and racial disparities in the clinical manifestations of atopic dermatitis [5]. Mutations in the filaggrin gene have a strong correlation in contributing to the susceptibility of developing atopic dermatitis [6]. The filaggrin gene is specifically responsible for the keratinization of skin, and impairment in this process results in skin barrier dysfunction [7]. Part of a complex of genes known as the epidermal differentiation complex which encodes proteins responsible for the development of the epidermal structure and barrier, studies have found that mutations in the filaggrin gene are present in roughly 25–50% of patients with AD [4–9].

Various environmental factors also contribute to atopic dermatitis. As a result of impaired skin barrier, allergens and irritants have increased permeability and elicit greater epidermal inflammatory responses in patients with AD [10]. Common environmental factors that play a role in eliciting exacerbation of AD symptoms include air pollutants, environmental and dietary allergens, seasonal changes, temperature variation, and microbes [11, 12, 13].

There are many complications associated with atopic dermatitis including increased risk of infection and significant impacts on quality of life. Patients with AD are found to have greater prevalence of the S. Aureus bacteria which can lead to bacterial epidermal infections with common infections including impetigo, cellulitis, and abscesses [14]. Atopic dermatitis also leads to greater risk of developing viral infections such as eczema herpeticum [14]. Negative impacts to quality of life are a more common complication associated with atopic dermatitis. Common complications include severe pruritus leading to sleep deficits with other impacts to quality of life including psychosocial and mental health impacts [15].

While atopic dermatitis primarily affects the epithelial system, there are correlations between AD and morbidities with other systems of the body. Comorbidities associated with atopic dermatitis may arise due to related pathogenesis to AD or due to the effects of AD symptoms [16]. In this review, the relation between AD and other systems will be highlighted as the impact of lifestyle and modifiable factors on the manifestation of atopic dermatitis is explored.

### Lifestyle and Health

Lifestyle choices are one of the most important and arguably most governable factors that can influence one’s health. Throughout the years, many research studies have been conducted analyzing the effects of daily lifestyle habits regarding topics such as sleep, stress, and diet on maintaining general health. It has been found that patients with chronic illnesses and multimorbidities are more likely to be associated with a poorer lifestyle with habits such as poor sleep hygiene, poor stress management, physical inactivity, inappropriate diet and smoking [17, 18]. To address the correlation between poor lifestyle habits and poorer health, lifestyle medicine and altering lifestyle habits can be assessed.

Lifestyle medicine is a branch of medicine that focuses on preventing and managing disease symptoms through six pillars of beliefs: plant-based diet, appropriate physical exercise, appropriate sleep hygiene, stress management, avoidance of risky substances, and maintaining positive social connections [19]. By altering one’s lifestyle to include healthier habits, studies have shown that incorporation of lifestyle medicine ideals and lifestyle modifications can offer effective improvement and management of symptoms for patients suffering from chronic diseases [20]. For patients suffering from atopic dermatitis, lifestyle management is often analyzed as a method of treatment and relief in conjunction with conventional treatment options.

### Effects of Sleep

Proper sleep hygiene is an essential factor that helps restore and maintain the health of a variety of systems throughout the body. The pruritus nature of atopic dermatitis has been noted as a common cause for insomnia in patients and the development of worsened sleep quality have been shown to result in heightened atopic dermatitis conditions [21,22]. For individuals suffering from atopic dermatitis, a disturbance in sleep can cause a variety of physiological responses involving the immune system, thermoregulation of body temperature, and fluid balance that results in a flare up of AD symptoms [21,22].

One way in which sleep disorders have been found to cause atopic dermatitis flare ups is highlighted in the relation of sleep with the immune system. Studies have shown that a loss of sleep leads to a decreased release of natural killer cells and results in the skin to be more susceptible to infections. [21, 23]. A greater increase in the release of inflammatory cytokines also occurs due to loss of sleep which contributes to damaging of the skin barrier [21, 24]. Elevated cytokine levels lead to an increase in the pruritus nature of AD and coupled with the decrease of Prostaglandin E2 which assist in repairing the skin barrier and are released during sleep, further compromises skin barrier and ability for barrier damages to heal [21, 22, 25].

In relation to the immune system, another physiological response that is impacted by sleep is thermoregulation. During sleep, studies have shown that while there are variations in body temperature throughout the night, the general trend of the body’s core temperature is at its lowest during the evening [21, 26]. As sympathetic tone is decreased during sleep, vascular flow increases within the cutaneous layer resulting in heat dissipation through the skin and elevated cutaneous temperature [26, 27]. This increase in the temperature of the skin has been associated with magnified nocturnal pruritus and exacerbation of atopic dermatitis conditions [26].

During night time, the skin barrier is further compromised as a rise in transepidermal water loss (TEWL) is noted [26]. Not only has TEWL been found to naturally rise during the evening, but studies have shown that TEWL rates are even further increased in patients with AD and in individuals who have poorer sleep quality which patients with AD often report [28, 29]. Associated with TEWL increase, this physiological mechanism results in greater itch and decreased ability for repair to skin damage [28, 29].

### Effects of Stress

Stress is a major factor that has significant effects on the symptoms and manifestations of atopic dermatitis. Studies have shown that different types of stress including oxidative and psychological stress can play a role in both manifestation of atopic dermatitis and the triggering of AD symptoms [30].

Oxidative stress is defined as an imbalance between oxidants and antioxidants within the body where an inclination towards oxidant levels is present [31, 32]. Increase in reactive oxygen and nitrogen species coupled with a decrease in antioxidant defense results from a variety of factors including environmental factors, psychological factors, and from the effects of aging and disease. [32–35]. Oxidative stress poses negative effects to bodily functions by increasing proinflammatory cytokines [32, 33]. In relation to cutaneous effects, an increase in proinflammatory cytokines creating an inflammatory environment can lead to the development and worsening of atopic dermatitis through increased pruritus and delayed wound healing [32, 33]. In fact in patients with AD, oxidative stress levels have been found to be markedly increased [32, 36]. A study researching the relation between oxidative stress and chronic skin conditions has discovered biomarkers of oxidative stress to be significantly elevated in the urine of patients presenting with AD, with levels of magnitude mirroring the severity of presenting symptoms [32, 36].

Psychological stress has also been indicated as a significant contributor to AD with various types of psychological stress contributing to the manifestation of eczema symptoms [33, 37]. While the mechanism of how psychological stress impacts the nature of AD is not well known, studies have indicated the state of psychological stress causes prolonged activation of the hypothalamus-pituitary-adrenal axis resulting in markedly increased release of stress biomarkers, leading to decreased immunity, delayed wound-healing, and increased TEWL in AD patients [33, 38]. In patients with AD, studies have shown AD can conversely cause worsened psychological stress and decreased quality of life due to intense pruritus, poor body image, and decreased self-confidence [33, 37–40]. While there is significant correlation between AD and psychological stress, whether the manifestations of AD precede psychiatric conditions or psychiatric conditions precede the development of eczema is still relatively unclear [37].

### Effects of Diet

The link between diet and atopic dermatitis is another lifestyle factor that has been studied to better understand the association between dietary consumption and the triggering and manifestation of AD. In relation to atopic dermatitis specifically, common food allergens such as dairy, peanuts, eggs, and gluten have been associated with being common triggers for food triggered AD [41, 42]. Food triggered AD can manifest in immediate or latent reactions. AD symptoms that manifest immediately are often triggered by IgE mediated immune responses and results in the exacerbation of pruritus as well as flare ups of erythematous lesions [43]. In latent reactions, exacerbation of AD symptoms are delayed for hours to days after consumption of the food trigger [44].

For individuals who experience food triggered AD from dairy, peanuts, and eggs, studies have found that these individuals not only exhibit significantly higher levels of IgE antibodies overall, but also exhibit higher levels of specific IgE antibodies to proteins found in dairy, peanuts, eggs, and cat dander compared to individuals who do not present with AD [44]. Specifically for individuals who exhibit dairy allergies, a study examining the prevalence of AD in 100 individuals with cow’s milk allergy found that up to 71% were found to have coexisting atopic dermatitis reactions [44]. In individuals experiencing AD flare ups as a result of consuming gluten, a significant link between gluten intolerance and atopic dermatitis has been explored. The inability to properly absorb gluten and the result of inflammation from gluten consumption has been shown to lead to cutaneous manifestations such as AD [44,45].

The relationship between the gut microbiome and atopic dermatitis has also been investigated to further examine the association of food triggers and AD. In many studies that have been conducted, results have shown significant correlation between the diversity of an individual’s gut microbiome with their tendency to develop eczematous lesions [45–48]. These studies suggest that with a decreased diversity of gut microbiome, individuals suffer from dysfunctional immune responses increasing the susceptibility to allergic reactions such as AD [46–48]. Furthermore, this imbalance in gut microbiota leads to greater sensitivity to certain foods, increasing food intolerances which contributes to the exhibition of food triggered AD [49].

### Effects of Alcohol

Alcohol has been known to cause negative influences on various bodily systems, most commonly known to pose negative effects on the liver. While not as well known, alcohol consumption has been indicated to have negative impacts to the skin and contributes to increasing the risk of the manifestations and exacerbation of various skin diseases. Alcohol has been found to influence the skin under various mechanisms which include alcohol’s effect on gut microbiota, increasing inflammation, vascular permeability, and promoting oxidative stress [50–54].

Multiple studies have shown that alcohol consumption has been linked with creating imbalanced gut microbiomes although the exact mechanism as to how this imbalance is induced is not well understood [55, 56]. The alcohol induced change in the gut microbiome has been found to negatively affect gut integrity which further results in impairment of the immune system and increased release of proinflammatory cytokines [56]. Chronic exposure to alcohol can also lead to additional tissue injury which results in greater inflammation [54, 56]. Furthermore, the metabolism of alcohol has also been shown to result in increased oxidative stress as the process of metabolizing alcohol generates reactive oxygen species [54]. The generation and release of reactive oxygen species is greatly elevated under inflammatory conditions [54]. The ability of alcohol to increase microvascular permeability further contributes to the inflammatory reaction to consuming alcohol as increases in vascular permeability leads to greater inflammation specifically in the skin [50, 53]. Analyzing the mechanisms in which alcohol affects the body, these effects mentioned have all been previously discussed to impact the triggering and manifestation of atopic dermatitis conditions.

In many analyses conducted on the relationship between alcohol and atopic dermatitis, the effect of alcohol consumption during pregnancy on the risk of the offspring developing AD is a popular topic. While it is not clear if there is a direct causation between maternal alcohol consumption and later on development of AD in their offsprings, many studies have demonstrated a correlation that indicates alcohol consumption during pregnancy as a probable risk factor to developing eczema in the child’s life. A study conducted by Linnenberg et al., had shown that the risk of atopic dermatitis development in offsprings was significantly impacted by maternal alcohol consumption and is dose dependent [57]. The results of the study showed that the greatest risk for AD development was seen when maternal alcohol consumption reached four or more drinks a week at 30 weeks gestation [57]. In a longitudinal study conducted by Carson et al., a significant association between alcohol consumption and atopic dermatitis development was demonstrated with the greatest risk of AD development to present within the first 7 years of the child’s life [58]. A study later conducted by Wada et al., further demonstrated the increased risk of atopic dermatitis where they found a positive association between maternal alcohol intake with increasing risk of atopic dermatitis within the first 5 years of life [59].

### Effects of Smoking Tobacco

Smoking has been indicated in multiple studies to be a contributing factor to atopic dermatitis. Tobacco smoke has been shown to pose many negative effects to an individual’s general health with many studies demonstrating the development of immune system dysfunction and chronic inflammation as a result of cigarette use [60, 61]. The direct effects of tobacco smoke on the skin has been researched with studies indicating the elevated oxidative stress that results from smoking, and the impairment of the immune system, negatively impacts the skin’s protective barrier resulting in the development of various skin conditions [60, 62, 63, 64]. Discussing the effects of smoking on atopic dermatitis specifically, many studies have been conducted to explore the relationship between smoking and AD, with various conditions including active smoking or second hand exposure to smoke, different age ranges, and maternal exposure to smoke being analyzed.

Comparing the effects of direct smoke exposure to secondhand smoke exposure, studies have shown that both types of exposure to tobacco smoke result in a positive association with the development of AD [60]. In individuals who have direct exposure to tobacco smoke, studies have shown that there is a positive correlation in active smoking with AD development in both adults over the age of 18 and children under the age of 18 [60, 65]. Even in adults who do not exhibit a predisposition to atopic dermatitis, participating in direct smoking increases the prevalence of adult onset AD [66]. In individuals who are exposed to secondhand tobacco smoke, a positive association with AD was also found [60, 67]. However, in passive smoke exposure, studies have indicated that the risk of developing AD is greater in adults compared to children [60].

Maternal exposure to tobacco smoke and the risk of their offspring developing AD was also a popular topic that was explored. In many studies conducted, the results have shown that there is no significant association with maternal smoking and development of AD in offsprings [60]. However, other studies have produced conflicting results that have found an increase in risk of AD development in offsprings due to maternal tobacco exposure [68–70]. In some studies, it has even been found that while there was no significant association between active maternal smoking and AD risk in offsprings, a significant correlation was noted between passive maternal smoking, such as secondhand exposure, and risk of AD manifestation in offsprings [68, 71]. From these findings, it is still unclear whether there is an association between maternal exposure to tobacco smoke and development of AD in offsprings. More studies will have to be conducted to produce a more conclusive understanding of this topic.

### Effects of Temperature and Humidity

Various environmental factors play a role in managing atopic dermatitis symptoms, and conflicting results have been produced demonstrating the effects of temperature and humidity in individuals with AD [72]. Looking at the studies conducted in regard to the effects of temperature on AD, some studies have found that an increase in temperature results in reduced manifestation of AD [72–74]. In a paper published by Kantor et al., the article hypothesizes the relationship between higher temperatures with greater UV exposure to be the contributing factor to improved AD, as UV has been shown to have beneficial effects for eczematous conditions [72]. One proposed mechanism in which UV is beneficial to AD is it helps produce Vitamin D which is often found to be deficient in individuals who have atopic dermatitis, with levels of deficiency correlating with the severity of AD symptoms [75–77]. Other studies and research articles have produced contradictory results that demonstrated an increase in temperature to exacerbate AD symptoms. In a paper by Sargen et al., the article states that higher temperatures promote TEWL which is a major contributing factor to AD [78]. Warmer temperatures also lead to increased sweating which was seen in a study conducted by Langan et al., to be positively correlated with eczematous flare ups in individuals with AD [79]. Sweat produces inflammatory markers that are associated with dysfunction of the filaggrin gene which is positively correlated with the clinical manifestation of AD [79, 80].

Exploring studies that relate levels of humidity with AD prevalence, contrasting results were similarly found. In some studies, it has been found that under conditions of high humidity, protective measures against atopic dermatitis have been noted as the increase in humidity reinforces the skin’s barrier [73]. When the skin is exposed to environments where there is low humidity, studies have shown this leads to increasing TEWL resulting in dysfunction of the skin barrier and eczematous conditions [73, 81]. Lower humidity also results in increased production of inflammatory markers such as Interleukin-1 alpha, and keratinocyte proliferation which further exacerbates AD symptoms [73, 82, 83, 84]. In other studies, contradicting results have been produced demonstrating that an increase in humidity exacerbates AD instead as humid environments can contribute to increased sweating [78–80].

### Effects of Skincare and Cosmetic Products

There are many skincare products available that can produce beneficial results for individuals suffering from atopic dermatitis, but the ingredients in some products in the market can produce detrimental effects triggering AD flare ups and worsening AD symptoms. Two common ingredients found in skincare and cosmetic products that often induce AD symptoms include fragrance and parabens.

Fragrance is present in most skincare and cosmetic products and is often found to be a main trigger for skin reactions. The mechanism in which fragrances affect the skin is attributed to a magnified immune response [85]. In individuals more susceptible to allergies including those with atopic dermatitis, studies have shown that fragrance leads to an increase in cytokine release and increases inflammatory responses [85]. Studies have also shown that different sexes and age groups have different susceptibility patterns to fragrance triggered AD. Females have been found to be more susceptible to atopic dermatitis reactions towards fragrances compared to males which studies have hypothesized is due to the increased exposure females tend to have to products with fragrances [86–89]. Younger populations also tend to have increased exposure patterns to fragrances compared to older populations which leads to greater rates of fragrance triggered AD in the younger population [87].

Besides fragrance, the use of parabens in skincare and cosmetic products have also been shown to induce AD flare-ups. Parabens are a common preservative used to prevent the growth of bacteria and fungi that is found in most skincare and cosmetic products including makeup, body cleansing products, and even oral hygiene products [90, 91]. The mechanism in how parabens trigger AD is still not well known, though studies have shown that parabens act as endocrine disrupting compounds and can modulate the immune system through modifying cytokine and IgE production [92, 93]. Similarly to fragrance, the effects of parabens on the susceptibility of AD vary depending on age ranges. In many studies, it has been shown that paraben exposure during early childhood and prenatal exposure leads to greater risk for AD development, specifically early-onset atopic dermatitis, due to exposure during the maturation of the immune system [94–96]. Different from the effects of fragrance, studies have shown conflicting results regarding the effects of parabens on AD prevalence among different sexes. In a study conducted by Vindenes et al., females have demonstrated to possess higher levels of parabens in their urine due to greater exposure to personal care products, leading to greater susceptibility to paraben triggered AD [97]. In contrast, in a study conducted by Arafune et al., despite different exposure patterns to paraben-containing products that are observed between females and males, no significant difference was observed in the effect of parabens on AD [95].

### Preventive Lifestyle Modifications for Atopic Dermatitis

Analyzing the effects of different lifestyle factors that can impact atopic dermatitis, many studies have explored lifestyle modifications that can be utilized to manage AD symptoms. In response to the effects of stress on AD, participating in de-stressing activities such as listening to music, breathing exercises, physical activity, and meditation have been shown to significantly reduce stress, anxiety, and depression which can manage the effect of stress on AD [98, 99]. Diet modifications can also be made in order to manage AD symptoms. In a study conducted by Dhar et al., out of 100 individuals tested, significant improvement in AD was observed when certain foods including dairy, peanuts, eggs, seafood, and beans were eliminated from their diet for 3 weeks [100]. Other studies have also suggested significant improvement in AD symptoms through limiting the consumption of sweets [43, 101]. As alcohol consumption and tobacco use have both been associated with increasing the severity of AD, cessation of these activities may result in improvement of AD [66, 102]. In terms of temperature and humidity effects on AD, there is still much debate regarding which climatic factors are more beneficial for symptom management so it is unclear whether avoiding one climate over the other would prove to be a significant modification for AD management. Skincare and cosmetic modifications that can be implemented include avoiding products that contain fragrances and parabens, and to incorporate products that contain antioxidants and anti-inflammatory properties [103]. Products that include prebiotics have also been shown to improve AD symptoms and reduce pruritus through restoring the skin microbiome and barrier [104]. Topical pharmaceutical creams and treatments can also be utilized to manage AD flare-ups with common options including corticosteroids, non-steroidal agents, anti-inflammatory drugs, and calcineurin inhibitors [105–107]. Through practicing modifications and utilizing treatments to reduce the symptoms of AD, sleep hygiene can also be improved. Through improving sleep, studies have shown greater restoration to the skin barrier and improvement to AD conditions [108, 109].

## Conclusion

The research shows that lifestyle factors can play a significant role in the clinical manifestation and triggering of atopic dermatitis symptoms. The literature shows lack of sleep, psychological and oxidative stress, and certain foods including dairy, eggs, peanuts, and gluten, can all contribute to the exacerbation of AD. Alcohol use and tobacco use both contribute to worsening AD, and in cases where maternal smoking and alcohol consumption is occurring, the risk of AD development in offsprings can be increased. The effects of temperature and humidity on atopic dermatitis is still unclear with studies producing contradictory results in regard to which conditions provide beneficial or detrimental effects to individuals diagnosed with AD. Personal care products containing fragrances and parabens can also worsen atopic dermatitis. Studies have shown that due to variations in exposure, females and younger populations tend to be more susceptible to fragrance triggered AD. Younger populations also tend to be more susceptible to paraben triggered AD compared to older populations. Overall, these findings implicate the importance of considering lifestyle factors when assessing and treating atopic dermatitis. Along with other treatments available, lifestyle modifications can serve to be significant treatment options, and can play a contributory role to improving the quality of life of individuals with atopic dermatitis.

## Figures and Tables

**Figure 1:**
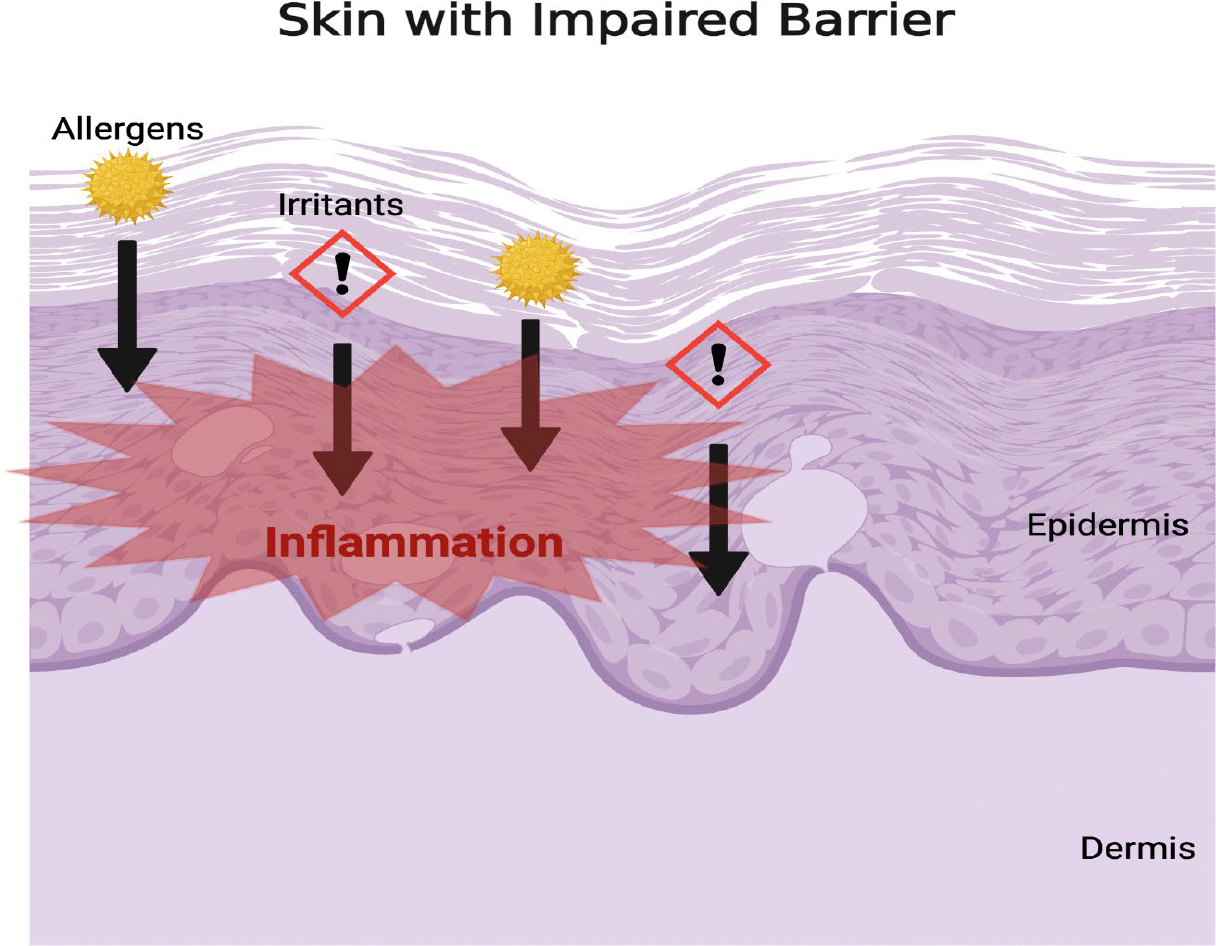
Dysfunction of the skin barrier allows for increased permeability of allergens and irritants leading to cutaneous inflammation.

**Figure 2:**
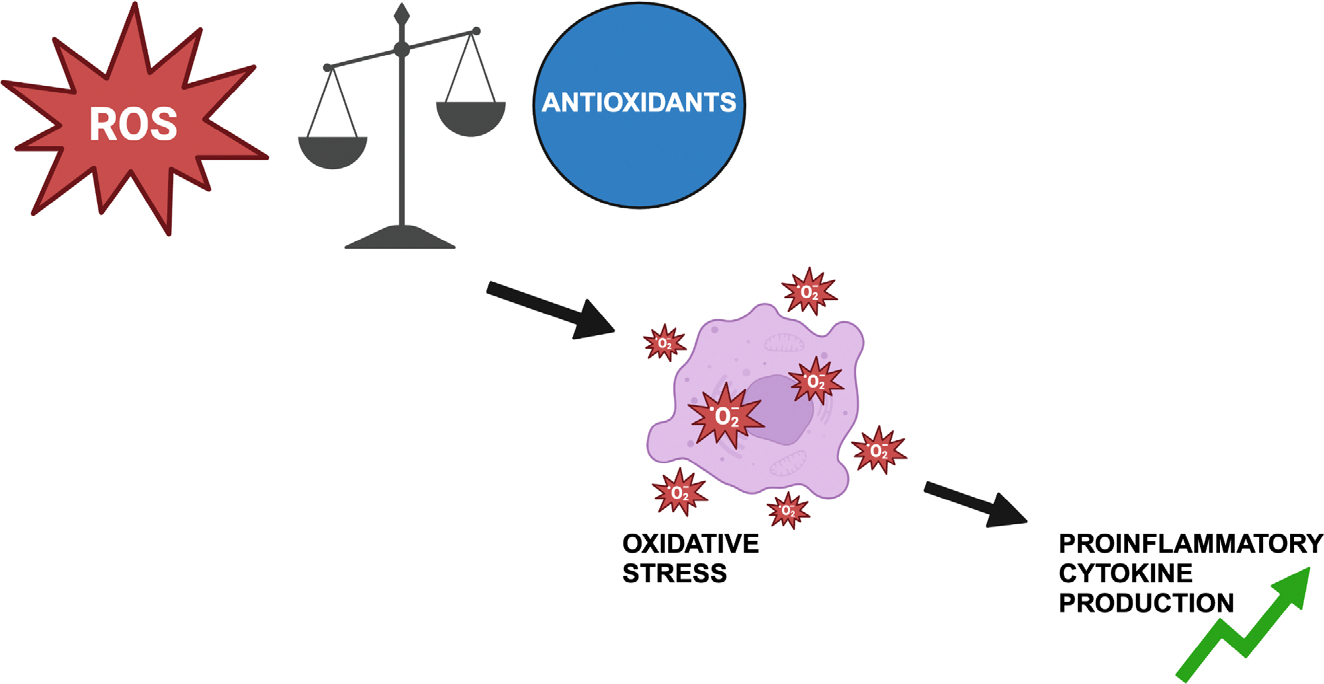
Imbalance in oxidants (reactive oxygen species, ROS) and antioxidants leads to oxidative stress which results in an increase in proinflammatory cytokine production. A greater inflammatory environment worsens atopic dermatitis symptoms.

**Figure 3:**
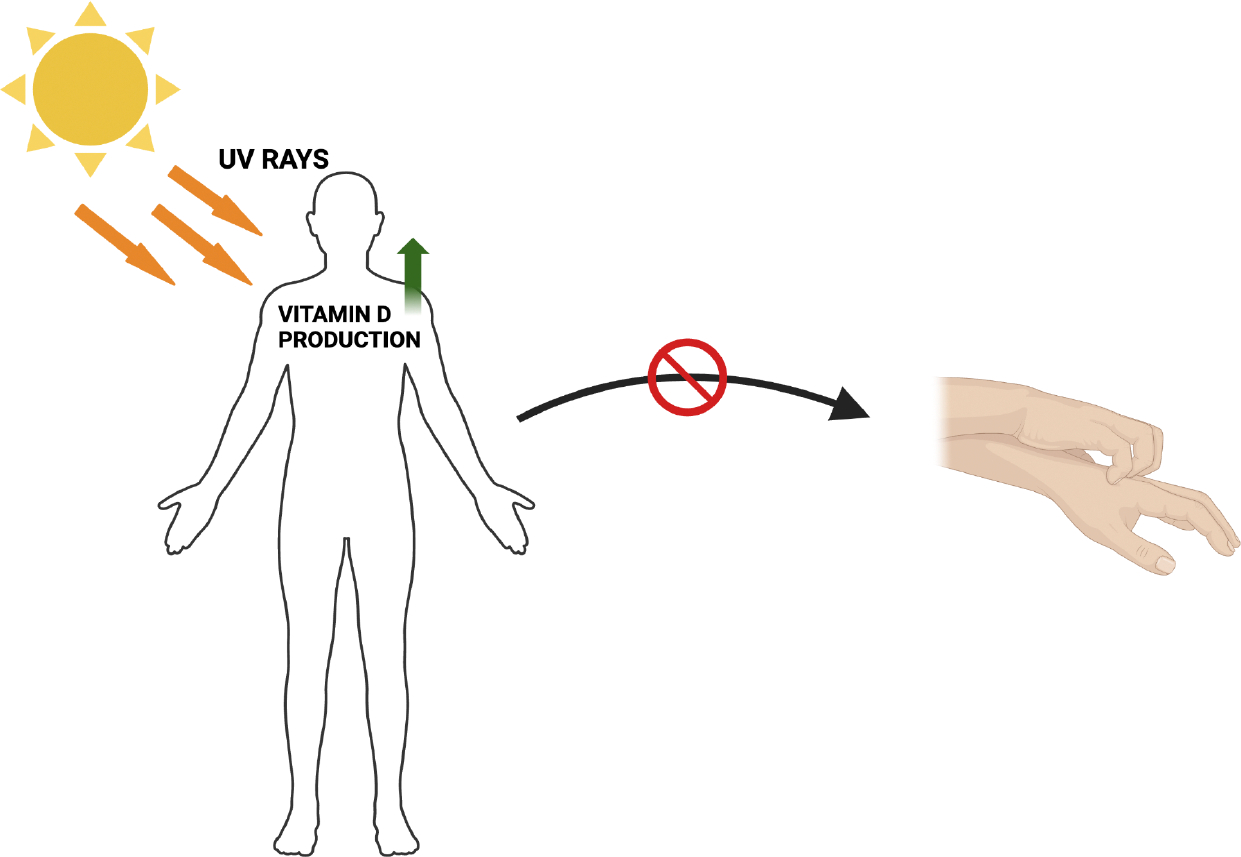
Greater exposure to ultraviolet (UV) rays due to warmer weather is hypothesized to improve atopic dermatitis symptoms through increasing Vitamin D production. Research suggests a link between Vitamin D deficiency and atopic dermatitis.

## Major Key Points and Outstanding Questions

- Lifestyle habits promoting inflammation are strong triggers for atopic dermatitis.
- Lifestyle modifications that protect and restore the skin barrier are important in preventing the manifestation of atopic dermatitis.
- Lifestyle changes that promote overall health are beneficial for managing eczema.
- How attainable is using lifestyle modifications to manage atopic dermatitis for individuals of low socioeconomic backgrounds?
- Is the usage of lifestyle modifications alone without traditional eczema treatments enough to provide significant long-term improvement to atopic dermatitis?
- How do certain genetic factors impact the effects of lifestyle factors and modifications on atopic dermatitis?
- What benefits are there to using lifestyle modifications over traditional eczema treatments?
- What differences causes the stark variations in how temperatures and humidity levels affect the atopic dermatitis conditions of different individuals?
